# Antidiabetic and Free Radicals Scavenging Potential of *Euphorbia hirta* Flower Extract

**DOI:** 10.4103/0250-474X.73921

**Published:** 2010

**Authors:** S. Kumar, R. Malhotra, D. Kumar

**Affiliations:** Institute of Pharmaceutical Sciences, Kurukshetra University, Kurukshetra-136 119, India

**Keywords:** Alloxan, antidiabetic, *Euphorbia hirta*, free radicals, mice, nitric oxide, superoxide

## Abstract

The present study was carried out to evaluate antidiabetic and *in vitro* free radicals scavenging effects of flower extract of *Euphorbia hirta*. The ethanolic and petroleum ether extracts (250 and 500 mg/kg) were orally tested for 21 days in alloxan induced diabetic mice and blood glucose level was measured with glucometer. Administration of extract resulted in significant reduction in serum cholesterol, triglycerides, creatinine, urea, alkaline phosphatase levels but high density lipoprotein levels and total proteins were found to be increased after treatments. Free radicals scavenging effect of ethanolic extract was also evaluated by various antioxidant assays, including 1, 1-diphenyl-2-picryl hydrazyl free radical scavenging activity, superoxide anion radical scavenging, nitric oxide scavenging, and reducing power assay. It was compared with standard antioxidants compounds such as butylated hydroxyl anisole and ascorbic acid. All the extracts showed antioxidant activity in all the tested methods.

Diabetes mellitus is a metabolic disease characterized by hyperglycemia resulting from defects in insulin secretion, insulin action, or both. It is well documented that chronic hyperglycemia of diabetes is associated with long-term damage, dysfunction, and eventually the failure of organs, especially the eyes, kidneys, nerves, heart, and blood vessels[1]. Synthetic antidiabetic agents can produce serious side effects and they are not suitable for use during pregnancy[2]. In view of the adverse effects associated with the synthetic drugs and as plants are safer, cheaper and much effective, conventional antidiabetic plants can be explored[3]. Furthermore, after the recommendation made by WHO on diabetes mellitus, investigations on hypoglycaemic agents from medicinal plants have become more important[4].

*Euphorbia hirta* (Euphorbiaceae), commonly known as *Dudhi* is an annual hairy plant. It is abundant in waste places along the roadsides and open grasslands. It is native to India and Australia[5]. The *E. hirta* have been reported to contain alkaloids, saponins, flavonoids, tannins phenolic acids and amino acids[6]. Traditionally, it is used in treatment of gastrointestinal disorders, bronchial and respiratory diseases, kidney stones, diabetes and in conjunctivitis. It also exhibits antipyretic, analgesic, antibacterial, anxiolytic, anthelmintic, antifertility, antispasmodic, antifungal, and antiinflammatory activities[7–9]. Our literature survey revealed that there is no experimental evidence of antidiabetic effect of the plant. Therefore, the present study has been planned to investigate how the ethanolic and petroleum ether flower extracts of *E. hirta* influences lipid parameters in alloxan induced diabetic mice.

All standard kits were purchased from Erba diagnostics Mannheim Gambh, Germany. Blood glucose level was measured using Elegance glucose meter (CT-X10) of Convergent Technologies, Germany. Alloxan was purchased from Loba Chemie pvt. Ltd. Mumbai, India. All other chemicals used in the study were of analytical grade.

Flowers of *E. hirta* were collected in the month of September-October, 2008 from campus of Kurukshetra University, Kurukshetra, India and were identified at the Department of Botany, Kurukshetra University, Kurukshetra, India. A voucher specimen of the plant is preserved in the herbarium of the Faculty of Pharmaceutical Sciences, Kurukshetra University (No. IPS/KUK/E-1/2009).

The flowers were washed with water and dried in shade. The dried flowers were powdered by using dry grinder and passed through sieve. This powder was packed into soxhlet apparatus and extracted successively with petroleum ether (60-80°) and ethanol (yield 26.83 and 24.74% respectively). All the extracts were dried at 45°in rotary evaporator to produce a semisolid mass and stored in airtight containers in refrigerator below 10°.

Albino mice of either sex, weighing about 30-35 g were used in the experiment. Animals were maintained under standard environmental conditions i.e. ambient temperature of 22±2 °and at 45–55% relative humidity for 12 h, each of dark and light cycle and fed with a standard pellet mice diet obtained from Ashirwad Industries, Chandigarh, India and water was supplied *ad libitum*. All the studies were conducted in accordance with the Animal Ethical Committee of the University.

Mice were made diabetic by a single intraperitoneal injection of freshly prepared alloxan (150 mg/kg i.p.) in sterile saline. Twelve days after alloxan injection, animals shown blood glucose level >140 mg/dl were considered as diabetic and used for the study. In the experiment, mice were divided into seven groups of six mice each.

Group I (Normal healthy control) received only vehicle (Tween 80, 5% v/v). Group II (Diabetic control) received vehicle only. In group III and IV diabetic mice received petroleum ether flower extract 250 and 500 mg/kg respectively). In group V and VI diabetic mice received ethanol flower extract (250 and 500 mg/kg respectively). Group VII was treated with glibenclamide (10 mg/kg).

The mice were given extracts for 21 days once daily by oral route using an intragastric tube. Blood glucose levels were measured using blood glucose test strips with elegance glucometer (Frankenberg, Germany) at random on initial, seventh, fourteenth, and twenty first day by taking blood samples from tail vein puncture under light ether anesthesia[10].

After blood glucose estimation on day 21, whole blood was collected by cardiac puncture under mild ether anesthesia from mice. Serum cholesterol, triglycerides, HDL, VLDL and LDL were also evaluated in normal and alloxan induced diabetic mice. The total cholesterol is measured using diagnostic kits, Boehringer Mannheim, Germany. Total cholesterol and triglycerides were determined by the method of Rifai *et al*.[11] HDL was measured by the method of Burstein *et al*.[12] The VLDL cholesterol was calculated using the formula (TG/5) mg/dl. The serum LDL cholesterol was estimated by the method of Friedwald *et al*.[13]

Serum creatinine, urea, alkaline phosphatase and total proteins levels were also evaluated in normal and alloxan induces diabetic mice. Serum urea and creatinine were assayed by the method of Tomas,[14,15]. Total proteins[16] and alkaline phosphatase were assayed by the method of Wilkinson *et al*.[17]

Antioxidant activity of *E. hirta* (ethanolic flower extract) was evaluated by following methods: DPPH free radical scavenging activity[18], superoxide radical scavenging assay[19,20], nitric oxide scavenging activity[21], and reducing power assay[22].

All the values of body weight, blood glucose and biochemical estimations were expressed as mean±standard error of mean (SEM) and comparison between the groups was made by student t- test. A valve of *p* < 0.001 was considered significant.

Antidiabetic effect of the extracts on the blood glucose levels of diabetic mice is shown in Table 1. Daily treatment of flower extracts for three weeks led to a dose dependent fall in blood glucose levels. Maximum effect seems to reach after 15 days of treatment and remains constant in third week. Administration of alloxan led to elevation of blood glucose levels, which was maintained over a period of three weeks. As shown both extracts induced significant (*p*<0.001) antidiabetic effects in dose-dependent fashion when compared to the control group.

**TABLE 1:** LONG TERM EFFECTS OF *E. HIRTA* EXTRACTS ON THE BLOOD GLUCOSE LEVELS IN NORMAL AND DIABETIC MICE

Groups	Blood glucose level (mg/dl)			
	Initial day	Day 7	Day 14	Day 21
Normal control	72.25±0.89	73.5±1.0	73.75±0.86	75.5±1.58
Diabetic control	185±2.88	186.2±1.7	189.25±1.25	192.5±1.73
Petroleum ether extract (250 mg/kg)	193.75±5.6*	175±5.8	162.7±3.1**	118.75±4.4**
Petroleum ether extract (500 mg/kg)	175.5±1.4*	152.5±1.4	140.2±2.9**	110.5±1.7**
Alcoholic extract (250 mg/kg)	172.7±0.8	121±1.2**	105.5±1.8**	86.2±0.5**
Alcoholic extract (500 mg/kg)	182.2±5.7	133.5±3.7**	124.5±2.7**	88.2±1.2**
Glibenclamide (10 mg/kg)	194.75±2.84	156.5±5.95**	116.5±5.24**	83.75±4.5*

Each value is the mean ±SEM. of 6 mice in each group

* *p*<0.05

** *p*<0.001

After 21 days, serum lipid profile was measured and it was found that serum cholesterol and triglyceride levels were decreased (*p*<0.01) by both the extracts and glibenclamide as compared to diabetic control but HDL level was found to be increased after treatments (Table 2). Other biochemical parameters such as serum creatinine, urea and alkaline phosphatase levels were found to be decreased whereas total proteins were found to be increased after treatments (Table 3).

**TABLE 2:** EFFECT OF CHRONIC EXPOSURE TO *E. HIRTA* EXTRACTS ON LIPID PROFILE

Groups	Cholesterol	Triglycerides	HDL Cholesterol	VLDL Cholesterol	LDL Cholesterol
Normal control	159.58±5.8	82.42±5.1	35±1.9	16.48±1.5	97.52±2.1
Diabetic control	257.83±14.6	250.62±12.7	30±1.2	50.12±1.4	177.71±7.4*
Pet. ether extract (250 mg/kg)	182.9±11.8	199±5.9	35.6±2.1	39.8±2.5	107.5±1.5
Pet. ether extract (500 mg/kg)	178.7±4.7*	188 ±4.7	39.2±1.4*	37.6±2.1	101.8±3.7*
Ethanol extract (250 mg/kg)	150.4±6.4	134±2.1	49.6±1.8	26.8±0.1*	84.3±3.2*
Ethanol extract (500 mg/kg)	146.3±5.9	131±1.9	50.2±2.1	26.2±2.7*	80.8±1.8
Glibenclamide (10 mg/kg)	120.16±5.7*	102±6.5*	64.52±1.9	20.4±2.8*	35.24±5.4*

Values are expressed as mg/dl, Each value is the mean±SEM of 6 mice in each group

* *p*<0.01

**TABLE 3:** EFFECT OF CHRONIC EXPOSURE TO *E. HIRTA* EXTRACTS ON OTHER BIOCHEMICAL PARAMETERS

Groups	Creatinine (mg/dl)	Urea (mg/dl)	Alkaline phosphatase	Total proteins (g/dl)
Normal control	0.61± 0.1	24.24 ±1.8	119.46±3.7	7.1±2.3
Diabetic control	1.53±0.1	72.00±2.4	351.40±6.2	4.3±4.62
Pet. ether extract (250 mg/kg)	0.79±0.6	50.46±3.9	270.2±5.9	5±0.3*
Pet. ether extract (500 mg/kg)	0.84±0.1	46±1.0*	240.6±6.4	5.3±2.3
Ethanolic extract (250 mg/kg)	0.59±0.5	42.2±1.4	151.2±4.9	4.8 ±1.3
Ethanolic extract (500 mg/kg)	0.64±0.1	32.7±1.7*	147.9 ±9.7	5.9±1.9*
Glibenclamide (10 mg/kg)	0.42±0.1*	30.00±3.2*	110.27±3.9*	8.4±1.4*

Each value is the mean±SEM of 6 mice in each group

* *p*<0.01

Normal healthy control was found to be stable in their body weight but diabetic mice showed reduction in the body weight. In this study, the decrease of body weights was diminished by the extract treatments after 14 days of treatment (Table 4).

**TABLE 4:** EFFECT OF *E. HIRTA* EXTRACTS ON THE BODY WEIGHT IN NORMAL AND DIABETIC MICE

Groups	Change in body weight			
	Initial day	Day 7	Day 14	Day 21
Normal control	27.3±1.9	27.96±1.5	31.11±3.8	28.59±1.1
Diabetic control	30.3±1.2	26.53±3.2	28.95±3.2	27.2±2.4
Petroleum ether extract (250 mg/kg)	25.6±1.8	24±1.7	21.7±2.0	25.6±3.2*
Petroleum ether extract (500 mg/kg)	26.7±2.0	25.2±1.9	23.4±1.8	26.7±2.0*
Alcoholic extract (250 mg/kg)	27.3±2.0	24.6±1.7	22.5±1.7	27.3±2.4
Alcoholic extract (500 mg/kg)	28.8±1.7	26.7±1.5	24.5±1.5	28.8±2.1*
Glibenclamide (10 mg/kg)	26.2±1.8	27.7±2.0*	29.89±2.2	30.46±1.9*

Each value is the mean±SEM of 6 mice in each group

* *p*<0.01

Both extracts of flower of *E. hirta* have significant antioxidant activity compared to other well characterized, standard antioxidant systems. Free radical scavenging potential was assessed against DPPH. The scavenging effect of alcoholic extract on the DPPH radical was 50.2%, at a concentration of 250 μg/ml. The reductive capabilities of extract were compared with ascorbic acid and BHA. The extract showed dose dependent reducing power. In the nitric oxide scavenging study, crude extract was checked for its inhibitory effect on nitric oxide production. The percentage inhibition of superoxide generation by 250 μg/ml concentration of extract was measured as 66.12%. The antioxidant activity of extract and standard compounds were compared by using specific *in vitro* methods (Table 5).

**TABLE 5:** ANTIOXIDANT PROFILE OF *E. HIRTA* FLOWER EXTRACT

Sample	Sample conc. (μg/ml)	DPPH radical scavenging activity (% inhibition)	Superoxide anion scavenging activity (% inhibition)	Percentage scavenging of nitric oxide	Reducing power activity^a^ (absorbance)
Alcoholic extract	250	50.2±1.7*	66.12±1.5*	34.6±2.1*	0.540
Ascorbic acid	250	95.44±1.4*	69.16±1.6	39.86±2.1	0.292
BHA	250	88.05±1.0*	67.74±3.1*	30.89 ±2.7*	0.248

a Increased absorbance indicates increased reducing power All values represent Mean±SEM

* *p*<0.01

Traditional plant medicines are used throughout the world for treatment of various diseases and disorders. Worldwide, over 1200 species of plants have been recorded as traditional medicine for diabetes. Herbal drugs are prescribed widely because of their effectiveness, less side effects and relatively low cost[23]. Therefore, investigation on such agents from traditional medicinal plants has become more important.

*Euphorbia prostrata*, the plant of same genus has been reported to have hypoglycemic effect[24]. Keeping in view of this and traditional uses, the ethanolic and petroleum ether extracts of E. hirta flowers were investigated for its antidiabetic activity and long-term effects on the body weights. Significant reduction of blood glucose levels is observed in alloxan induced diabetic mice treated with *E. hirta* flower exract (*p*<0.001).

The repeated administration of Euphorbia hirta extract for a period of 21 days resulted in a significant decrease in lipid parameter levels of various tissues when compared to the diabetic control. It is not known whether *E. hirta* has a direct effect on lipids or the present hypolipidemia is achieved due to controlled hyperglycemia.

Different studies have shown that Diabetes mellitus is associated with the increased formation of free radicals and decrease in antioxidant potential. In both insulin dependent and insulin independent diabetes, there is increased oxidative stress[25]. So, free radical scavenging and antioxidant effect may be responsible for its antidiabetic effect. It is possible that extract exert its effect by causing hypoglycemia. The exact mechanism of antidiabetic activity is still unclear but it may be due antioxidant and free radical scavenging effect of the plant and presence of flavanoids, tannins and other phenolic compounds in the extracts.

Thus, our study shows that oral administration of *E. hirta* flower extracts in alloxan diabetic mice showed antidiabetic effects. The extracts also exhibit *in vitro* antioxidative effect. Further phytochemical and pharmacological investigations are needed to isolate and identify the active constituents responsible for the activity.
